# Deubiquitination and stabilization of programmed cell death ligand 1 by ubiquitin‐specific peptidase 9, X‐linked in oral squamous cell carcinoma

**DOI:** 10.1002/cam4.1675

**Published:** 2018-07-10

**Authors:** Wu Jingjing, Guo Wenzheng, Wen Donghua, Hou Guangyu, Zhou Aiping, Wu Wenjuan

**Affiliations:** ^1^ Department of Laboratory Medicine Shanghai East Hospital Tongji University School of Medicine Shanghai China; ^2^ Key Laboratory of Cell Differentiation and Apoptosis of The Chinese Ministry of Education Shanghai Jiao Tong University School of Medicine Shanghai China; ^3^ Department of Oral and Maxillofacial surgery Hospital of Stomatology Tongji University Shanghai China

**Keywords:** oral squamous cell carcinoma, programmed cell death ligand 1, programmed cell death protein 1, ubiquitin‐specific peptidase 9, X‐linked

## Abstract

**Background:**

The immune checkpoint protein programmed cell death ligand 1 (PD‐L1) binds to PD1 to promote tumor cell escape from the killing effect of the immune system. However, there are few studies on the regulatory mechanisms of PD‐L1 in tumors. Although PD‐L1 has been reported to undergo ubiquitination in some cancers, its regulatory mechanisms in oral squamous cell carcinoma (OSCC) are unclear. Therefore, we aimed to investigate this phenomenon.

**Methods:**

We examined the expression and function of USP9X and PD‐L1 in human oral keratinocytes (HOK) and OSCC cell lines (HN4 and HN30) as the control and relevant cancer cells using western blotting, immunoprecipitation, immunohistochemistry (IHC), T‐cell‐mediated tumor cell killing assay, and liquid chromatography‐mass spectrometry.

**Results:**

Programmed cell death ligand 1 was highly expressed in OSCC by the regulation of the ubiquitin‐proteasome pathway. Furthermore, we discovered that ubiquitin‐specific peptidase 9, X‐linked (USP9X) could be combined with PD‐L1 to induce its deubiquitination and stabilize its protein expression in OSCC.

**Conclusion:**

Our data indicate that USP9X deubiquitinates and stabilizes PD‐L1. Suppressing the expression of USP9X blocks tumor cell growth. The results provide a theoretical basis for USP9X as a therapeutic target.

## 1 INTRODUCTION

Head and neck squamous cell carcinoma (HNSCC) is one of the 10 most common cancers worldwide. Oral squamous cell carcinoma (OSCC) is the most common malignant tumor of the head and neck.^1^, ^2^ Despite advances in multimodal therapeutic strategies including surgery, radiation, and chemotherapy, the 5‐year survival rate remains less than 20% in patients with advanced conditions.^3^, ^4^ Clarification of the pathogenesis is important to verify novel specific targets and identify more effective therapeutic strategies for OSCC.

M2 macrophages increase in number in patients with higher grades of OSCC. The tumor microenvironment might be immunosuppressive, while the M2 tumor‐associated macrophage phenotype might contribute to the further development of OSCC.^5^ In addition, the immunosuppressive effect of OSCC is related to the high expression of the programmed cell death ligand 1 (PD‐L1).^6^, ^7^ PD‐Ll is an important immunological checkpoint protein that binds to programmed cell death protein 1 (PD1) expressed on immune cells, such as activated T cells, by inhibiting the activation and proliferation of T cells.^8^

Programmed cell death ligand 1 expression levels in tumor cells have been reported to be positively correlated with tumor stage, metastasis, and recurrence rate.^9^ The expression of PD‐L1 is regulated by a variety of factors, such as signal transducer and activator of transcription (STAT), nuclear factor kappa B (NF‐κB), and nuclear factor of activated T cells (NFAT).^10^, ^11^ Li et al^12^ reported that glycogen synthase kinase 3β (Gsk3β) promotes the phosphorylation‐dependent proteasome pathway degradation of PD‐L1. In addition, ubiquitin‐specific protease COP9 signalosome 5 (CSN5) induced by NF‐κB p65 is required for tumor necrosis factor alpha (TNF‐α)‐mediated PD‐L1 stabilization in breast cancer cells. CSN5 inhibits the ubiquitination and degradation of PD‐L1.^13^ These observations show that posttranslational modification plays an important role in the stability of PD‐L1.

In the present study, we investigated the role of PD‐L1 in OSCC development using western blotting, immunoprecipitation, immunohistochemistry (IHC), T‐cell‐mediated tumor cell killing assay, and liquid chromatography‐mass spectrometry (LC/MS). Ubiquitin‐specific peptidase 9, X‐linked (USP9X) inhibited the ubiquitination and degradation of PD‐L1, thereby mediating the immune escape of OSCC. USP9X participates in a variety of normal biological functions of mammals.^14^ In addition, USP9X is involved in regulating tumor cell apoptosis, proliferation, adhesion, and other biological processes.^15^ USP9X is expressed abnormally in nonsmall cell lung cancer, breast cancer, melanoma, head and neck SCC, and other human malignancies.^16^, ^17^, ^18^, ^19^ USP9X regulates the ubiquitination of its target protein by ubiquitin‐specific protease activity, thereby affecting the expression level.^20^ Therefore, USP9X has become a potential target for tumor therapy. Here, we report that USP9X inhibits the growth of OSCC cells by deubiquitinating and stabilizing PD‐L1 and may serve as a therapeutic target.

## 2 MATERIALS AND METHODS

### 2.1 Cell lines and reagents

The cell lines used in this study were human oral keratinocytes (HOK) as control cells and human OSCC cells (HN4 and HN30) from tongue carcinoma and OSCC with associated metastasis. All cell lines were provided by the Shanghai Key Laboratory of Stomatology, Shanghai Ninth People's Hospital, Shanghai Jiao Tong University School of Medicine. Antibodies to USP9X and ubiquitin were from Santa Cruz Biotechnology (Santa Cruz, CA, USA), while those to hemagglutinin (HA) and Flag were purchased from Cell Signaling Technology (Beverley, MA, USA). Lipofectamine 2000 was purchased from Invitrogen (Carlsbad, CA, USA).

### 2.2 Cell culture and generation of stably transfected cells

Stably transfected OSCC cells and control HOK cells were maintained in Dulbecco's modified Eagle's medium (DMEM, Hyclone, UT, USA) supplemented with 10% (v/v) heat‐inactivated fetal bovine serum (FBS; Gibco BRL, Waltham, MA, USA) at 37°C in a humidified atmosphere of 5% CO_2_. To establish stable transfectants with knockdown of USP9X, HN4 cells were transfected with human USP9X‐specific short hairpin RNA (shRNA) and cloned into the pGIPz lentiviral empty vector. Scrambled lentivirus vector (shUSP9X‐NC; Thermo Fisher Scientific, Waltham, MA, USA) was used as the control. The target sequences were shUSP9X‐3#, 5ʹ‐CTTAAATCCTCATTGCAAA‐3ʹ, and shUSP9X‐5#, 5ʹ‐TGCTTGTGTCTTTAGATGA‐3ʹ. Cells (5 × 10^5^/well) were seeded into a six‐well plate and grown to 60%‐80% confluence. The cells were transfected with equal concentrations of shUSP9X‐3#, shUSP9X‐5#, or shUSP9X‐NC in 2 mL lentiviral supernatant containing 8 g/mL polybrene, followed by incubation for 6 hours at 37°C. Then, the cells were cultured in fresh DMEM supplemented with 10% FBS for 48 hours. Stable clones were selected and incubated with puromycin (1.5 g/mL) for 2 weeks. The shRNA transfection efficiency was determined by examining the expression of USP9X using western blot analysis.

### 2.3 Soft agarose

For this experiment, 2% agarose and DMEM + 10% FBS (1:3, final concentration 0.7%) were placed in wells of a 24‐well plate (0.5 mL/well) and left for 10 minutes at 4°C to enable the agarose to solidify. HN4 and HN30 cells (500 cells/50 μL medium) were added to the medial interlayer, and 0.5 mL of a mixture of 2% agarose and DMEM + 10% FBS (1:6, final concentration, 0.35%) was placed on the cells with Matrigel (1:30). The plate was incubated at 37°C in a 5% CO_2_ incubator, and the images were acquired and statistically analyzed after 2 weeks.

### 2.4 Immunoprecipitation and LC/MS

Cells were pretreated with 5 μmol/L MG132 (a specific, potent, reversible, and cell‐permeable proteasome inhibitor) for 4 hours and lysed in 1 mL radioimmunoprecipitation assay (RIPA) buffer (50 mmol/L Tris‐hydrochloride [HCl, pH 7.4], 150 mmol/L sodium chloride [NaCl], 1 mmol/L ethylenediaminetetraacetic acid [EDTA], 1% Triton X‐100, 2 mmol/L N‐ethylmaleimide [NEM], and broad‐spectrum protease inhibitor cocktail [1:100]). The cell lysate was incubated with anti‐Flag M2 beads at 4°C overnight, and then the beads were washed five times with RIPA containing 2 mmol/L NEM. The immunoprecipitated proteins were denatured with loading buffer, and the samples were subsequently analyzed using immunoblotting or visualized with Coomassie brilliant blue (G250). Bands that were different between HN4 and HOK in the latter staining assay were further analyzed using MS.

### 2.5 IHC

Tumor tissue specimens were obtained from 20 patients with primary OSCC during surgical resection in the Department of Oral and Maxillofacial Surgery, Hospital of Stomatology, Tongji University, between 2015 and 2017. The patients provided their consent before the tissue collection, and they did not receive any radiotherapy or chemotherapy before surgery. Adjacent normal oral epithelial tissues from the same patient that showed normal USP9X and PD‐L1 expression were used as their respective controls. Subsequently, the tissue specimens were serially sectioned, processed using standard protocols, and then examined to observe the relationship between USP9X and PD‐L1 in their expression and positioning. The experimental steps have been described previously.^21^

### 2.6 T‐cell‐mediated tumor cell killing assay

T‐cell‐mediated tumor cell killing assay was performed according to the manufacturer's protocol (Promega, Madison, WI, USA). To analyze the killing of tumor cells by T‐cell inactivation, we cocultured USP9X‐knockdown tumor cells and control cells with peripheral blood mononuclear cells in 96‐well plates. T cells were activated with CD3/CD28 antibody‐conjugated beads. According to the instructions in the CytoTox 96^®^ Non‐Radioactive Cytotoxicity Assay, signals were measured and quantified at 490 nm after the addition of lysate buffer, substrate solution, and stop solution. Alternatively, to visualize the surviving tumor cells at the endpoint, after a 24‐hours coculture of tumor cells and T cells in 12‐well plates, the wells were washed twice with phosphate‐buffered saline to remove T cells. The surviving tumor cells were fixed and stained with crystal violet solution.

### 2.7 Additional assays

The western blotting and cell clonogenic assay are outlined in Data S1.

### 2.8 Statistical analysis

The data were analyzed and plotted using the Prism software (GraphPad, San Diego, CA, USA). Comparisons between two groups were made using an unpaired Student's *t* test. Differences were considered significant at *P* < 0.05.

## 3 RESULTS

### 3.1 PD‐L1 protein is overexpressed in OSCC cells

**Figure 1:**
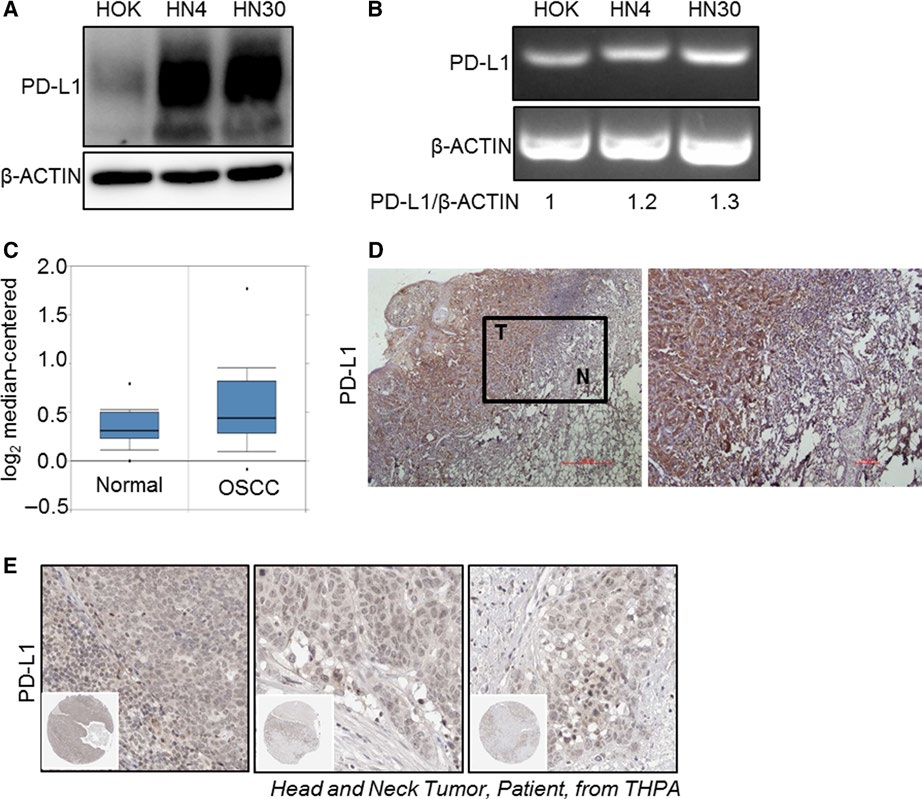
Protein level expression of programmed cell death ligand 1 (PD‐L1) was high in oral squamous cell carcinoma (OSCC). A, Expression of PD‐L1 in OSCC (HN4 and HN30) cell lines was high compared with that in normal human oral keratinocyte (HOK) cells. B, mRNA expression of PD‐L1 between OSCC (HN4 and HN30) and oral normal cell line (HOK) showed no significant difference. C, mRNA expression of PD‐L1 from Oncomine database was not different between patients with OCSS and normal individuals. D, Immunohistochemistry (IHC) showed expression of PD‐L1 in tumor and paracarcinoma tissue. E, IHC data from The Human Protein Atlas (THPA) database showed PD‐L1 was highly expressed in OSCC samples

Tumor cells avoid the immune system mainly because of aberrantly expressed immune checkpoint proteins on the surface of tumor cells, especially PD‐L1. While research on PD‐L1 in a variety of tumors has been very thorough,^22^ there are relatively few studies on OSCC. Presently, we found that the protein levels of PD‐L1 in HN4 and HN30 cells were significantly higher than that in HOK cells (Figure 1A). However, the changes in the mRNA level of PD‐L1 were not significant (Figure 1B). This trend in mRNA expression was also verified in the Oncomine database (http://www.oncomine.org, Figure 1C). IHC staining showed that PD‐L1 immunopositivity in OSCC cells was higher than that in paracarcinoma tissue (Figure 1D). Moreover, we searched for results of partial IHC staining in The Human Protein Atlas (THPA) database (http://www.proteinatlas.org) concerning the expression of PD‐L1 in patients with oral squamous cell cancer. PD‐L1 was generally highly expressed in OSCC tumors (Figure 1E). Taken together, these results suggest that PD‐L1 was aberrantly expressed in OSCC tumors, especially at the protein level.

### 3.2 Overexpressed PD‐L1 in OSCC is regulated by deubiquitination

**Figure 2:**
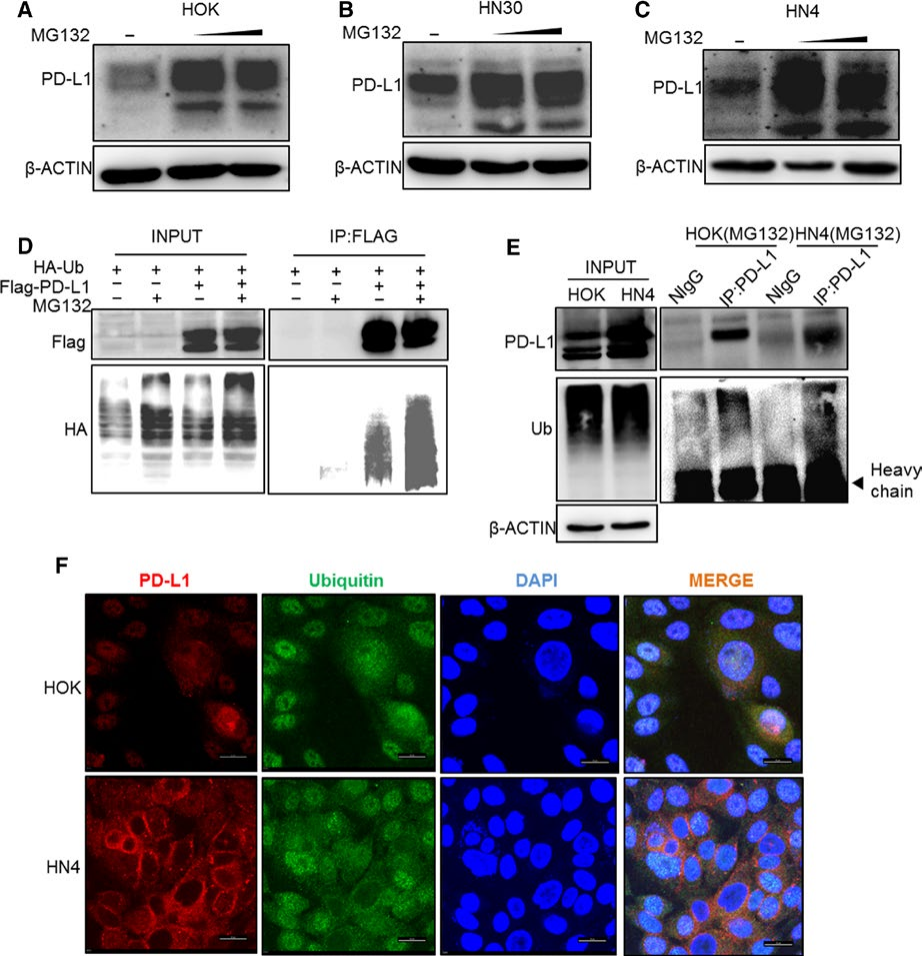
Overexpressed programmed cell death ligand 1 (PD‐L1) was regulated by deubiquitination. A‐C, Protein level of PD‐L1 in oral squamous cell carcinoma (OSCC, HN4, and HN3) and normal human oral keratinocyte (HOK) cell lines treated with MG132 (10 and 20 μmol/L for 12 h). D, Interaction between exogenous PD‐L1 and ubiquitin in HEK293T cells. HEK293T cells overexpressing Flag‐PD‐L1 and HA‐ubiquitin were treated with MG132. E, Interaction between endogenous PD‐L1 and ubiquitin in HN4 and HN30. Cells were immunoprecipitated with PD‐L1 antibody, and ubiquitin expression was measured. F, Immunofluorescence indicated that PD‐L1 was overexpressed in HN4 cells and colocalized with ubiquitin. Scale bar, 20 μm

Based on the above results, we hypothesized that PD‐L1 might undergo protein posttranslational modification, especially ubiquitination, by proteasome pathway degradation. As protein degradation is accompanied by ubiquitin K48 chain ubiquitination, we analyzed PD‐L1 protein expression in the presence of MG132 in HOK cells. MG132 induced PD‐L1 protein accumulation (Figure 2A). The increase in protein expression also occurred in HN4 and HN30 tumor cells treated with MG132 (Figure 2B,C). To further verify the ubiquitination of PD‐L1, we designed and performed exogenous and endogenous immunoprecipitation experiments. Ubiquitin, which was combined with PD‐L1, increased after MG132 treatment of HEK293T cells overexpressing Flag‐PD‐L1 and HA‐ubiquitin (Figure 2D). Similarly, ubiquitin of the endogenous PD‐L1 also increased in HOK and HN4 cells treated with MG132 (Figure 2E). Moreover, endogenous ubiquitin and PD‐L1 proteins strongly interacted as observed in HOK and HN4 cells in the immunofluorescence assay (Figure 2F). Taken together, these results indicated that overexpression of PD‐L1 in OSCC cells was mostly due to the regulation of deubiquitination.

### 3.3 Deubiquitinase USP9X interacts with PD‐L1 in OSCC cells

**Figure 3:**
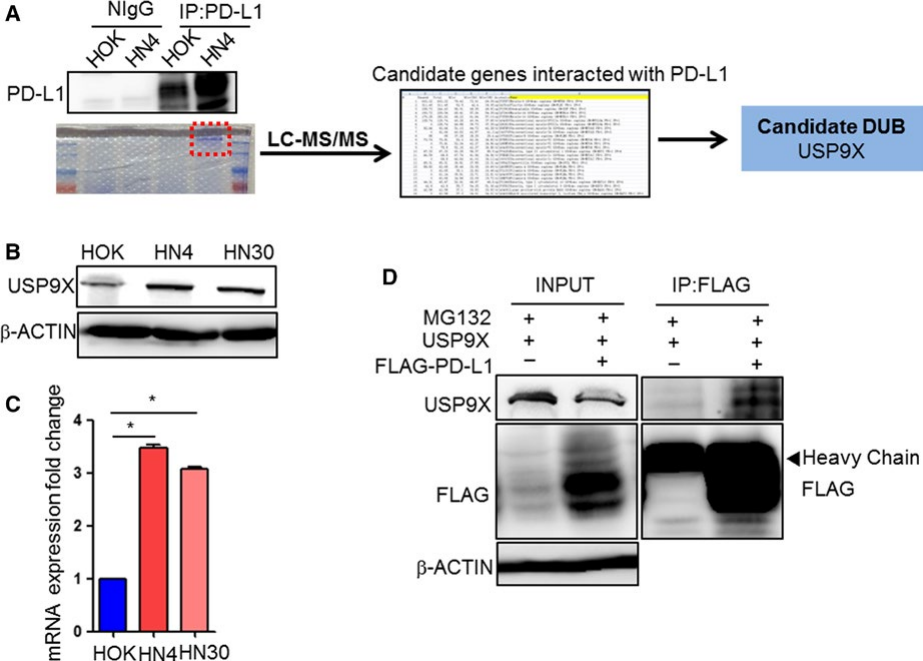
Deubiquitinase ubiquitin‐specific peptidase 9, X‐linked (USP9X) interacted with programmed cell death ligand 1 (PD‐L1) in oral squamous cell carcinoma (OSCC). A, Flowchart of analysis of proteins bound to PD‐L1 using liquid chromatography‐tandem mass spectrometry (LC‐MS/MS). Flag‐PD‐L1 was immunoprecipitated using Flag antibody‐conjugated (M2) agarose beads. USP9X was selected as candidate binding protein in 682 identified PD‐L1 binding proteins. B, Western blot analysis of deubiquitinating enzyme USP9X expression in OSCC (HN4 and HN30) cell lines and normal human oral keratinocyte (HOK) cell line. C, Quantitative reverse transcription‐polymerase chain reaction (qRT‐PCR) analysis of USP9X in HOK, HN4, and HN30 cell lines. *p < 0.05. D, Interaction between exogenous PD‐L1 and USP9X in HEK293T cells. HEK293T cells overexpressing Flag‐PD‐L1 and USP9X were treated with MG132. Cells were immunoprecipitated with Flag‐M2 beads, and expression of USP9X was measured

We found that the expression of PD‐L1 was regulated by its ubiquitination in OSCC cells. Thus, the regulatory mechanism appeared to be particularly important. To explore this, we first analyzed the protein interaction with PD‐L1 using LC‐MS after immunoprecipitation (Figure 3A). Among the 682 identified proteins, USP9X was determined to be the candidate deubiquitinase (Figure 3A). Moreover, both protein (Figure 3B) and mRNA (Figure 3C) levels of USP9X in HN4 and HN30 cells were significantly increased, compared with that in HOK cells. Interestingly, the exogenous PD‐L1 and USP9X strongly interacted, as shown in the in vitro co‐immunoprecipitation experiment (Figure 3D). We further speculated from the above results that the combination of USP9X with PD‐L1 might increase the deubiquitination process of PD‐L1 and reduce its degradation, leading to protein accumulation.

### 3.4 USP9X stabilizes PD‐L1 by its deubiquitinase activity

**Figure 4:**
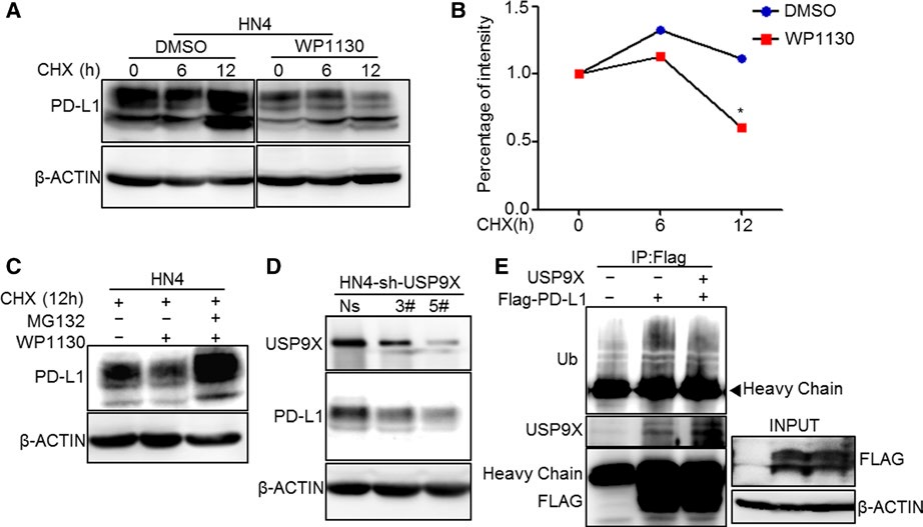
Ubiquitin‐specific peptidase 9, X‐linked (USP9X) stabilized programmed cell death ligand 1 (PD‐L1) by decreasing ubiquitination. A and B, Protein stability of PD‐L1 in HN4 cells. Cells were treated with 20 μmol/L cycloheximide (CHX) at indicated intervals with or without WP1130 (4 μmol/L for 12 h) and analyzed using western blotting. *p < 0.05. C, HN4 cells were treated with CHX for 12 h, and protein stability was measured using western blotting after treatment with or without MG132 and WP1130. D, Expression of PD‐L1 in USP9X‐knockdown HN4 cell was determined using western blotting (Ns, non‐specific knockdown plasmid). E, USP9X decreased ubiquitination of PD‐L1. HEK293T cells were transfected with Flag‐PD‐L1 and USP9X. Ubiquitin was analyzed after immunoprecipitation of Flag with or without USP9X

To confirm the deubiquitination function of USP9X in OSCC cells, we utilized WP1130, a deubiquitinase inhibitor that targets USP9X. Half‐life analysis using cycloheximide indicated that WP1130 reduced the expression of PD‐L1 as well as its protein half‐life in HN4 and HN30 cells, especially at the 12‐hour time point (Figure 4A,B and Figure S1A,B). HN4 cells treated with WP1130 showed decreased PD‐L1 protein levels, which were restored after treatment with MG132 (Figure 4C). This experiment showed that USP9X stabilized PD‐L1 by decreasing its ubiquitin level. Silencing USP9X in tumor cells further validated the correlation between PD‐L1 and USP9X. As shown in Figure 4D and Figure S1C, the expression of PD‐L1 was significantly decreased in HN4‐ and HN30‐knockdown USP9X cells compared to the control cells. Moreover, the ubiquitin protein bound to PD‐L1 was reduced to a certain extent in HEK293T cells overexpressing USP9X (Figure 4E and Figure S1D). The results further validated our hypothesis that the highly expressed USP9X increased and decreased the deubiquitination and ubiquitination of PD‐L1, respectively, leading to its accumulation in OSCC cell lines.

### 3.5 USP9X is critical in OSCC tumor growth

**Figure 5:**
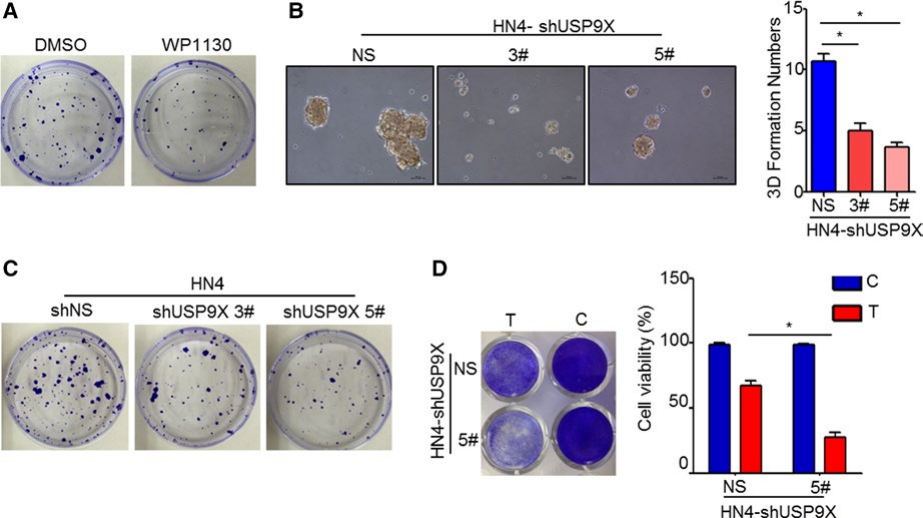
Ubiquitin‐specific peptidase 9, X‐linked (USP9X) was critical for oral squamous cell carcinoma (OSCC) tumor growth. A, Palate formation was significantly decreased in HN4 cells treated with WP1130 (4 μmol/L for 10 days). B, Soft agarose assay of USP9X‐knockdown HN4 cells (*P < 0.01). C, HN4 cells stably transfected with USP9X‐shRNA were seeded in six‐well plates at a density of 2000 cells/well and cultivated for 2 wk. Cells were then stained, photographed, and counted using microscopy. D, T‐cell‐mediated tumor cell killing assay in USP9X‐knockdown and control cells. Activated T cell and HN4 cells were cocultured in 96‐well and 12‐well plates for 24 h, respectively, and the surviving tumor cells were visualized at 490 nm or by crystal violet staining (*P < 0.05)

As USP9X deubiquitinated PD‐L1 in vitro, we sought to determine the role of USP9X in OSCC progression. We first examined the proliferation of tumor cells using a plate colony formation assay. The results indicated that proliferation of HN4 and HN30 cells treated with WP1130 was obviously suppressed (Figure 5A and Figure S2A). Moreover, the three‐dimensional colony formation was also decreased in both number and size in USP9X‐knockdown OSCC cell lines (Figure 5B and Figure S2B,C). As expected, the proliferation of the USP9X‐knockdown OSCC cell lines was reduced significantly (Figure 5C and Figure S2D). To validate the role of USP9X in PD‐L1 regulation, we performed a T‐cell killing assay (Figure 5D) and found that USP9X was functionally required for PD‐L1 regulation. The results suggest that USP9X not only can increase the level of PD‐L1 but also plays an important role in tumor growth.

### 3.6 USP9X is highly expressed in clinical OSCC samples

**Figure 6:**
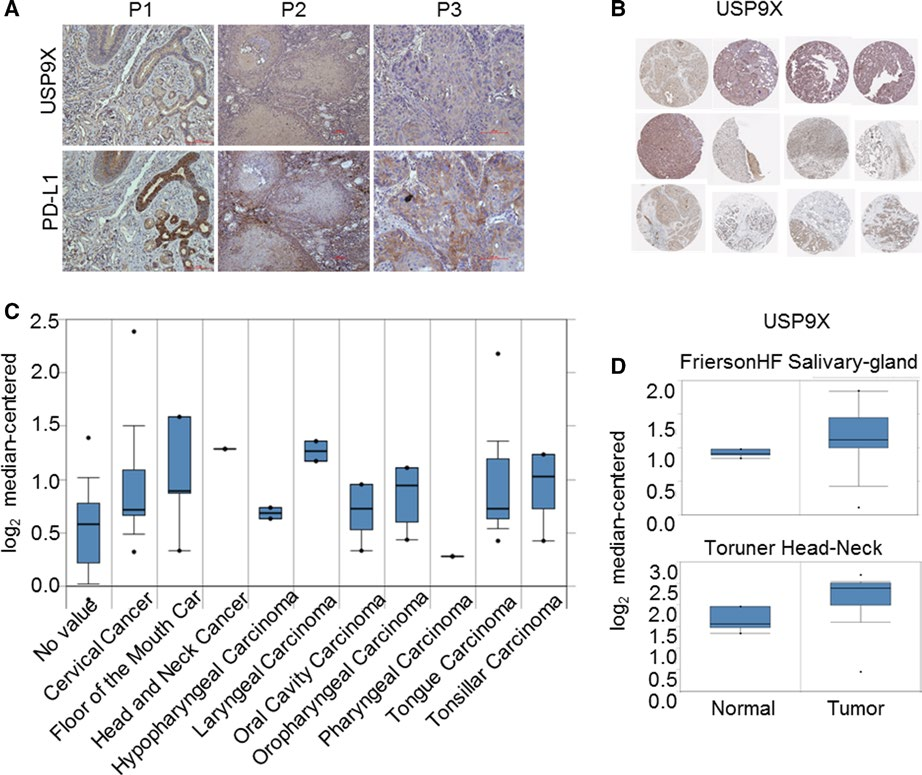
Ubiquitin‐specific peptidase 9, X‐linked (USP9X) was highly expressed in oral squamous cell carcinoma (OSCC) tissues. A, Immunohistochemistry (IHC) data showing expression of programmed cell death ligand 1 (PD‐L1) and USP9X in sequential tissue slices of several patients with OSCC. B, Expression of USP9X in patients with OSCC patients was generally high. IHC data were from The Human Protein Atlas (THPA) database (n = 12). C, mRNA data from Oncomine database showed USP9X was highly expressed in several head‐and‐neck‐related tumors. D, mRNA of USP9X was highly expressed in OSCC tumors compared to normal tissue

Next, we detected USP9X and PD‐L1 expression in sequential tissue slices from patients with OSCC using IHC (Figure 6A). PD‐L1 also appeared to be highly expressed in high USP9X expression areas. We used online data to further validate the expression of USP9X in samples from patients with OSCC. We selected 12 tissue samples from patients with OSCC from THPA (http://www.proteinatlas.org) and found that the expression of USP9X was generally higher in tumor than in normal tissues (Figure 6B). In addition, mRNA data from the Oncomine database (http://www.oncomine.org) showed that USP9X was also highly expressed in several head‐and‐neck‐related tumors (Figure 6C). In two specific OSCC databases, the level of USP9X in cancer was also higher than that in normal tissues (Figure 6D,E). Collectively, our results suggest that upregulation of USP9X in OSCC led to the deubiquitination of PD‐L1 and stabilized its expression, which enhanced its interaction with PD‐1 to escape T‐cell immune surveillance.

## 4 DISCUSSION

Our results clarify that USP9X induces PD‐L1 deubiquitination and stabilizes its protein expression in OSCC cells. PD‐L1 is expressed in cancer cells, leading to immune escape.^23^ However, little is known about the posttranslational protein modifications of PD‐L1 in cancer, especially in OSCC. Recent studies showed that inflammation increases PD‐L1 expression through CSN5, which reduces PD‐L1 ubiquitination and leads to its stabilization.^13^ CSN5 was identified as a PD‐L1‐interacting partner using MS analysis, confirming an association between PD‐L1 and CSN5 in breast cancer.^13^ We also determined the function of CSN5, USP15, and CSN8 in our system and found that they did not affect PD‐L1 protein expression and stability. There may be several reasons for this observation. First, the mechanism of the posttranslational modification of PD‐L1 protein may differ in various cancer cells, which might be related to the heterogeneity of tumor cells and their different microenvironments. Second, the NF‐κB pathway in OSCC cells is not in a state of sustained activation compared to normal oral cells. Therefore, CSN5, which depends on the NF‐κB pathway, might not important in OSCC, especially in the regulation of PD‐L1 expression. Taken together, these findings suggest that activated tumor signaling pathways vary in different tumor microenvironments.

Interestingly, USP9X reduced the ubiquitination of PD‐L1 and stabilized its protein expression. However, the mechanisms regulating the high expression of USP9X in OSCC are unclear. There are several classically activated pathways in OSCC, including hypoxia, human papillomavirus (HPV) infection, and Notch pathways.^24^, ^25^, ^26^ HIF‐1α overexpression has been associated with tumor cell growth and survival in head and neck tumors. Moreover, HPV is responsible for the rising proportion of OSCC and might induce the expression of oncogenes.^27^ Numerous expression and functional analyses have demonstrated that Notch1 plays a crucial role in the development and progression of OSCC.^26^ Presently, USP9X played an important role in OSCC, but whether the expression of USP9X in OSCC was affected by these activation pathways is unclear and is a major focus for our future studies.

In conclusion, we demonstrate that USP9X is highly expressed in OSCC and stabilizes PD‐L1 by reducing its ubiquitination. Importantly, the tumor formation of USP9X‐knockdown OSCC cells is significantly decreased. Hopefully, targeting PD‐L1 by blocking USP9X might be a potentially useful strategy to treat OSCC, especially metastatic tumors.

## CONFLICT OF INTEREST

The authors declare no conflict of interest.
