# Supplementary figures and images for: Deubiquitination and stabilization of programmed cell death ligand 1 by ubiquitin‐specific peptidase 9, X‐linked in oral squamous cell carcinoma
Source: Cancer Med. 2018 Jul 10;7(8):4004–11. doi: 10.1002/cam4.1675 (PMC6089178; doi:10.1002/cam4.1675)

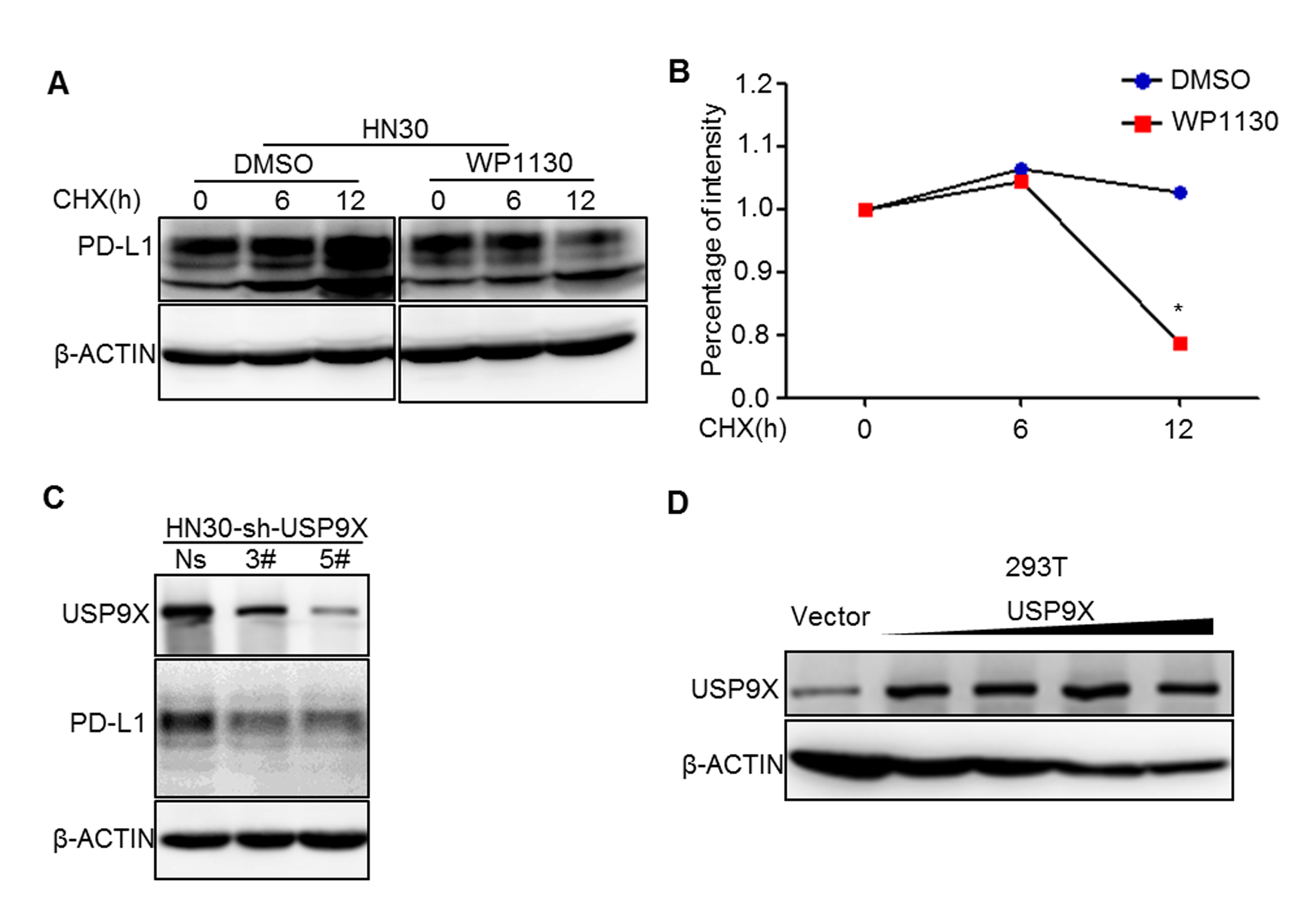

Supplement: Supplementary file 1 [file CAM4-7-4004-s001.tif]

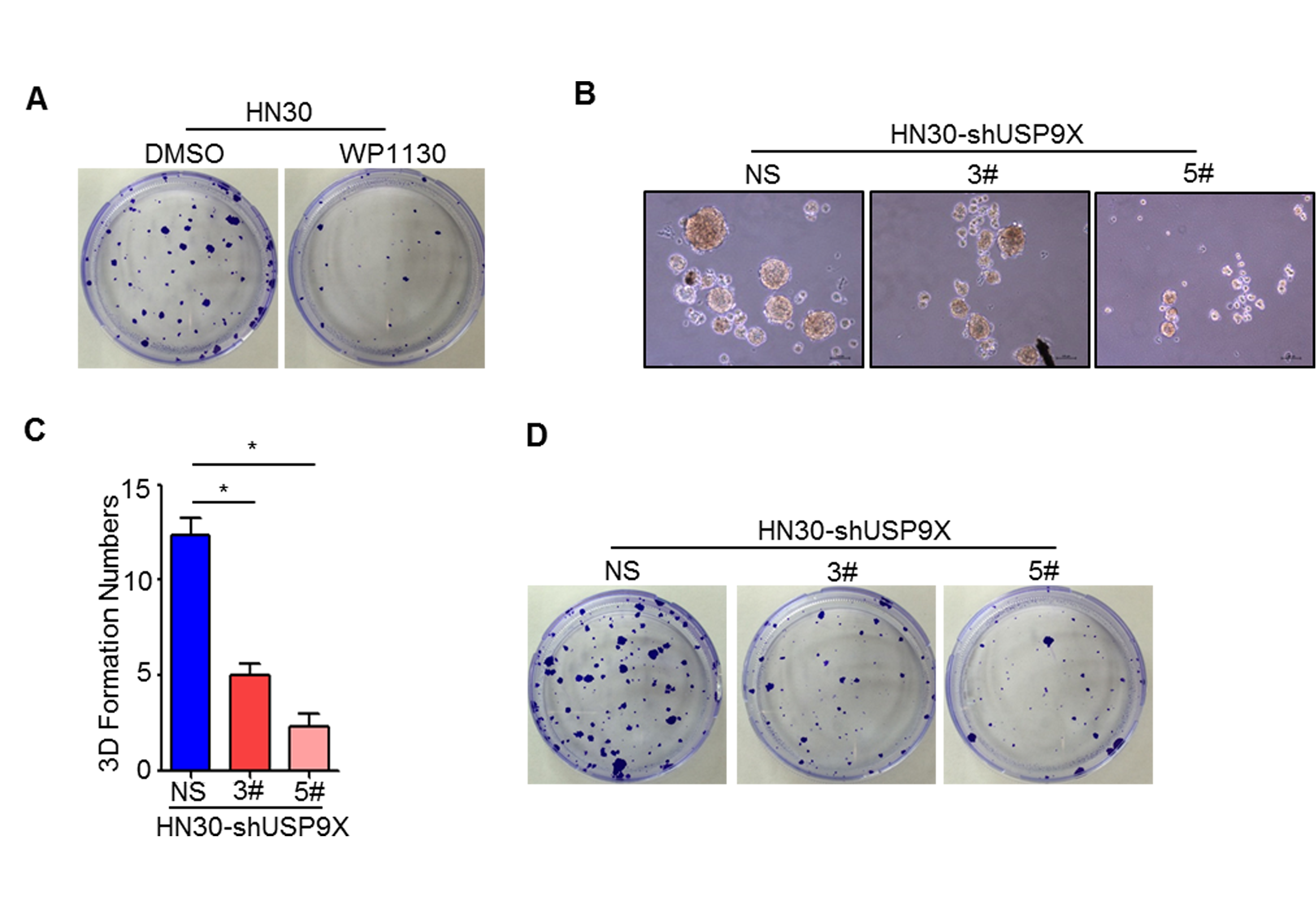

Supplement: Supplementary file 2 [file CAM4-7-4004-s002.tif]
