# Supplementary material for: Deubiquitination and stabilization of programmed cell death ligand 1 by ubiquitin‐specific peptidase 9, X‐linked in oral squamous cell carcinoma
Source: Cancer Med. 2018 Jul 10;7(8):4004–11. doi: 10.1002/cam4.1675 (PMC6089178; doi:10.1002/cam4.1675)
Supplement: Supplementary file 3 [file CAM4-7-4004-s003.docx]

**Data S1. Supplementary Materials and Methods**

*Western blotting*

Cells were washed with PBS and lysed with lysis buffer (50 mM Tris-HCl, pH 6.8, 100 mM DTT, 2% SDS, 10% glycerol). Cell lysates were centrifuged at 20,000 g for 10 min, and proteins in the supernatants were quantifed. Protein extracts were equally loaded onto 8–12% SDS-polyacrylamide gels, electrophoresed, and transferred to a nitrocellulose membrane (Bio-Rad). The blots were stained with 0.2% Ponceau S red to ensure equal protein loading. After blocking with 5% nonfat milk in PBS, the membranes were probed with antibodies. The signals were detected with a chemiluminescence phototope-HRP kit (Cell Signaling) according to the manufacturer’s instructions. As necessary, blots were stripped and reprobed with anti-β-actin (Calbiochem) or β-tubulin (Sigma-Aldrich) antibody as an internal control. All experiments were repeated three times.

*Cell clonogenic assay*

Cells were seeded into 6-well plates at a density of 2000 cells/well in 2 ml medium containing 10% FBS. Culture medium was changed every 3 days for 2 weeks. The cell clones were stained for 15 min with the solution containing 0.5% crystal violet and 25% methanol, followed by rinsing with tap water three times to remove excess dye. Colonies consisting of more than 50 cells were counted under microscope.
